# Deprescribing antipsychotics in patients with schizophrenia: findings from a specialized clinic

**DOI:** 10.1017/S0033291724001910

**Published:** 2024-10

**Authors:** Alexander Nøstdal, Rikke Hilker, Christina Halgren, Helene Speyer, Mette Ødegaard Nielsen, Jimmi Nielsen

**Affiliations:** 1 Unit for Complicated Schizophrenia, Mental Health Centre Glostrup, Copenhagen University Hospital – Rigshospitalet, Nordstjernevej, 2600 Glostrup, Denmark; 2 Department of Clinical Medicine, University of Copenhagen, Blegdamsvej 3B, Copenhagen, Denmark; 3 Mental Health Centre Copenhagen, Copenhagen University Hospital – Rigshospitalet, Lersø Parkallé 112, Copenhagen, Denmark; 4 Mental Health Centre Copenhagen, Copenhagen University Hospital – Rigshospitalet, Copenhagen Research Center for Mental Health – CORE, Gentofte Hospitalsvej 15, Copenhagen, Denmark

**Keywords:** adverse effects, antipsychotics, deprescribing, level of function, schizophrenia

## Abstract

**Background:**

While antipsychotic medication reduces the risk of relapse for patients with schizophrenia, high prevalence of adverse effects results in low adherence. Lower doses of antipsychotics have been associated with increased level of function but also with increased risk of relapse. This study presents findings from a specialized deprescribing clinic. In addition, we aim to identify clinical predictors for relapse.

**Methods:**

Patients diagnosed with schizophrenia were referred to the clinic, which offers a six-month guided tapering program. Antipsychotic dose was reduced by 10% every four weeks. Patients were monitored closely for symptom progression or decrease in level of function, with defined cut-offs prompting a pause in or cessation of dose reduction.

**Results:**

After 12 months, the antipsychotic dose was reduced from 404 (±320 mg) to 255 (±236 mg) chlorpromazine equivalent. Of the 88 patients included, 22 (27%) experienced relapse during the six-month tapering period, while 29 (37%) experienced relapse at the 12-month follow-up visit and nine patients were antipsychotic free. Patients who remained stable experienced a slightly increased level of functioning and markedly fewer side effects (*p* < 0.001). Following relapse, patients were clinically stabilized and showed an improved attitude toward antipsychotic medication. The predictive models were weak.

**Conclusions:**

We show that most patients undergoing guided antipsychotic tapering remained stable after one year and improved in level of function, while most patients who relapsed were quickly stabilized. Our inability to create strong predictive models could be due to limitations in the study design, warranting future studies exploring tapering of antipsychotics in patients with schizophrenia.

## Introduction

While the principles of deprescribing (Scott et al., 2015) are gaining traction in other medical fields, dose reduction or discontinuation of antipsychotic medication in patients with schizophrenia remains a controversial topic. Despite patients frequently requesting it, clinical guidelines on dose reduction are vague and weakly underpinned by evidence (Potla et al., 2023).

At the core of the controversy lies diverging opinions on the weight of risks associated with long-term treatment and the risks associated with relapse. Life-long maintenance treatment may worsen cognitive deficits, anhedonia, somatic morbidity, and premature mortality (Correll, Rubio, & Kane, 2018). In contrast, discontinuation has been associated with increased mortality in register-based data (Tiihonen, Tanskanen, & Taipale, 2018), and it has been argued that longer duration of antipsychotic treatment is associated with increased life expectancy (Correll et al., 2018), although these conclusions have been criticized and heavily debated (Taylor & Horowitz, 2020; Tiihonen, Taipale, & Correll, 2020; Whitaker, 2020). Rather than synthesizing these risks in binary recommendations, it should be kept in mind that shared decision making, where personal values and preferences are essential, is at the core of evidence-based practice (Mccormack & Elwyn, 2018). When prescribers and researchers debate this topic, the patient perspective is often neglected, undermining the process of shared decision making, and may result in lack of support and trust, often leading to non-adherence (Velligan, Sajatovic, Hatch, Kramata, & Docherty, 2017), where patients stop treatment without professional support.

Deprescribing is an approach where risks and benefits are continuously assessed in every patient, considerations are shared openly, and reduction can be initiated at any time the expected benefits do not outweigh the expected harms (Gupta, Cahill, & Miller, 2018). While discontinuation may be the goal of many, *dose reduction* has potential benefits as well. Some adverse effects, e.g. sedation, and cognitive and extrapyramidal symptoms, are associated with dose, while others, like weight gain and dyslipidaemia, are not dose dependent (Yoshida & Takeuchi, 2021). Several studies have shown better functioning and quality of life among patients receiving no or lower doses of antipsychotics (Harrow, Jobe, Faull, & Yang, 2017; Stürup et al., 2022b; Wils et al., 2017; Wunderink, Nieboer, Wiersma, Sytema, & Nienhuis, 2013), although a recent randomized trial found no difference in level of function in their interim analyses of the first two years when comparing maintenance treatment with dose reduction. Importantly, this study did not discriminate between stable patients and patients who relapsed (Moncrieff et al., 2023).

Relapse-prevention studies report a substantially lower risk of relapse in those who receive maintenance treatment (Beasley et al., 2003; Chen et al., 2010; Moncrieff et al., 2023). A recent meta-analysis (Højlund, Kemp, Haddad, Neill, & Correll, 2021) comparing relapse rates between standard dose and reduced dose found that low dose increased the relapse risk by 44%, and very low dose increased the risk by 72% in patients with multi-episode schizophrenia. However, most antipsychotic discontinuation studies have terminated long term treatment abruptly, which may lead to withdrawal effects confounding the relapse rate (Munkholm, Horowitz, & Moncrieff, 2022). Several naturalistic studies and randomized trials report that a subgroup of patients can discontinue antipsychotic medication without relapse (Beasley et al., 2003; Chen et al., 2010; Harrow et al., 2017; Moilanen et al., 2013; Wils et al., 2017), suggesting that discontinuation is indeed possible for some.

Identifying the subgroup least likely to relapse after dose reduction would be of clinical interest because it has the potential to predict outcome and thus prevent unnecessary relapses, as well as support deprescribing in individuals at low risk of relapse. Systematic reviews have identified several proposed predictors (Tani et al., 2018), but follow-up studies have shown low levels of replicability (Bowtell, Ratheesh, McGorry, Killackey, & O'Donoghue, 2018), possibly due to selection/attribution bias and confounding by unmeasured covariates. Another possible explanation is the conflation of prediction and causal modelling (Ramspek et al., 2021). As no predictors have yet been replicated, the decision to taper medication remains largely a matter of trial and error.

In this observational cohort study, we report the tolerability of dose reduction by describing the development of relapse over time and in relation to antipsychotic doses. Our primary aim is to examine and compare the level of function between patients remaining stable and those experiencing a relapse. We hypothesize an improvement in patients remaining stable during dose reduction, whereas a transitory drop in level of function is expected in patients experiencing a relapse. Our secondary aim is to compare the development in adverse effects, attitude toward medication, level of symptoms, and antipsychotic doses between stable patients and patients who relapse. Finally, we explore demographic and clinical predictors of the subgroup that did not tolerate dose reduction.

## Methods

### Study design

Based on requests from patients and relatives, the Danish health authorities established a novel specialized outpatient clinic in 2018 at the Mental Health Centre Glostrup in the Capital Region of Denmark (catchment population 1 822 659 (Statistics Denmark, 2018) to provide guided tapering of antipsychotics in patients diagnosed with schizophrenia. The Danish Data Protection Agency (file no.: 2012-58-0004, RHP-2018-003, 6144) approved the study and all participants provided written informed consent for their data to be collected and used for research purposes. After evaluating the study protocol, the Danish National Committee on Biomedical Research Ethics and the Danish Medicines Agency exempted the project from submitting a formal application because the decision to reduce or discontinue antipsychotic medication was independent of the research purpose.

Following a thorough baseline examination, the participants entered a six-month treatment program, where they were closely monitored for signs of relapse while the antipsychotic medication was tapered according to the initial plan defined by the treating physician together with the individual patient. In addition to weekly contact with the treatment team, the patients had consultations with the treating physician every four weeks. At the end of the six-month outpatient program, the patients were typically referred for continued treatment to the outpatient clinic or general practitioner who had originally referred them to the project. They were also invited to a follow-up session 12 months after inclusion.

### Assessment

Apart from demographic information and psychiatric history, patients were evaluated with clinical rating scales at baseline and monthly for six months. We used the Global Assessment of Functioning (GAF) to evaluate the level of functioning (American Psychiatric Association, 1987); the Positive And Negative Syndrome Scale (PANSS) for symptom severity and progression (Kay, Fiszbein, & Opler, 1987); and Udvalget for Kliniske Undersøgelser (Clinical Investigation Committee) Side Effect Rating Scale (UKU) for side effects (Lingjærde, Ahlfors, Bech, Dencker, & Elgen, 1987). Attitude toward antipsychotics was assessed at baseline and after six months using Drug Attitude Inventory–10-item version (DAI) (Ernst, Lindström, Nielsen, & Levander, 2012; Hogan, Awad, & Eastwood, 1983).

### Standardized guided tapering

The tapering of antipsychotics followed a standardized plan designed and aimed to reduce the antipsychotic dose at four-week intervals by an amount corresponding to 10% of the antipsychotic dose at time of enrolment. When possible, tablets were halved, or intervals increased to every second day, and for depot medication, the injected dose was decreased. However, commercially available tablet sizes or prefilled syringes for injection sometimes limited the precision of the tapering. In cases where this necessitated a reduction significantly larger than 10% of dose at enrolment, the intervals between dose reductions were increased accordingly, e.g. a 20%-reduction would result in eight weeks between dose adjustments, see examples of tapering regimens in online Supplement material. Symptom assessment was performed at four-week intervals, regardless of time between dose adjustments. In instances of antipsychotic polypharmacy, one of the drugs was selected for tapering, based on experienced adverse effects and the individual patient's preferences. Antipsychotic doses are described both as chlorpromazine equivalent (CPZeq) (Leucht et al., 2014; Rey, Schulz, Costa, Dick, & Tissot, 1989) and defined daily doses (DDD) (WHO, 2021).

We used PANSS to evaluate the development of symptoms and PANSS Score was one of the determining factors in terms of whether further tapering of antipsychotic medication was recommended. Cut-offs for moderate and severe symptom progression were set at a 10- and 15-point increase in total PANSS score, respectively. In the case of moderate symptom progression, patients were recommended to continue current medication dose until the next symptom assessment, while patients experiencing severe symptom progression were advised to increase their dose of antipsychotic medication (or in some cases, to switch to an alternative antipsychotic drug), and further attempts at tapering were stopped. We aspired to treat the participants according to the principles of shared decision-making: following the withdrawal protocol was entirely voluntary. In case of relapse, patients were recommended but never forced to increase antipsychotic medication dose.

In several instances, early warning signs, such as increased levels of anxiety or sleep disturbances, would prompt the patients to reconsider further tapering. In these cases, patients were encouraged to remain on the present dose despite no detectable symptom progression on PANSS. The possibility for further dose reduction was re-evaluated at the following assessment.

### Outcome definition

Outcome was grouped into patients remaining stable throughout the observation period and patients with severe worsening of symptoms, subsequently denoted from here on as ‘stable’ and ‘relapse’, respectively. Symptomatic remission was not an inclusion criterion, and this study defined relapse as a worsening of symptom load, significant negative effect on social functioning, being admitted to a psychiatric inpatient care facility, suicide attempts, or episodes of violence. This outcome was observed at 6 and 12 months.

### Statistics

The Kaplan–Meier method was used to construct survival curves, with relapse *v.* no relapse as status indicators. We included all participants who completed the study and participants who relapsed before their final examination, whereas participants who discontinued the study without known status where excluded. We constructed curves illustrating relapse status over time and relapse status in relation to dose in DDD at the time of relapse.

We used a paired *t* test to analyze changes in GAF scores from baseline to 6 and 12 months and the lowest GAF score in relation to a relapse. We employed repeated measures ANOVA to analyze the group × time interaction. The same methods were used to compare PANSS scores from baseline to six months. Values were log transformed for GAF and PANSS subscores.

We used the Wilcoxon signed-rank test to analyze changes in the DAI score, reported side effects in terms of UKU sub- and total score after six months, and antipsychotic dose (CPZeq) after 6 and 12 months. Analyses were performed for the whole group of patients and on each subgroup (i.e. stable and relapse after six months). We used the Mann–Whitney *U* test to examine the DAI score for between-group differences at baseline and after six months.

For the predictive analysis, backward logistic regression was performed with stable-relapse status at 6 and 12 months as the dependent variables. Based on findings from previous literature (Tani et al., 2018), age, age at diagnosis, duration of treatment, baseline medication dose (CPZeq), and GAF and PANSS subscores at baseline were entered as independent variables. Because age at diagnosis was missing for 29 patients and duration of treatment for 23 patients, the analysis was performed with and without these variables.

## Results

**Figure 1.**
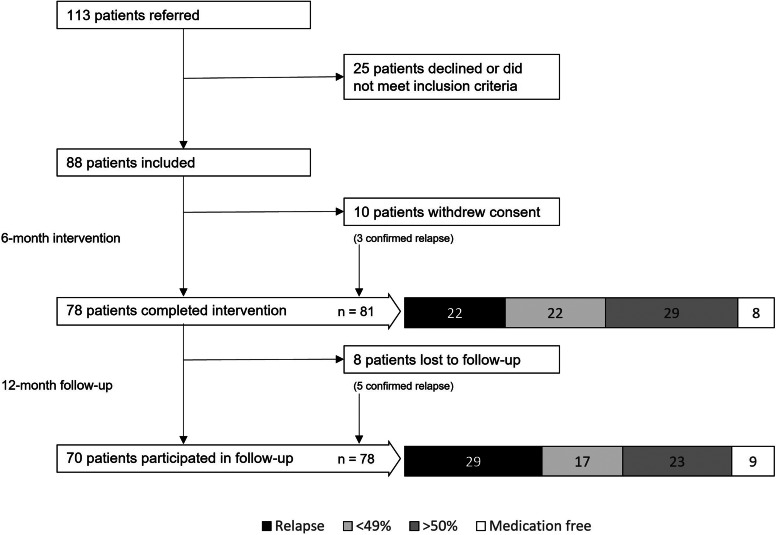
Study flowchart. Eight patients were without antipsychotic medication after six months. Three of these patients were back on antipsychotic medication six months later. A total of 9 patients were antipsychotic free 12 month after entering the project, since 4 patients had continued tapering in their regular outpatient setting.

Between May 2018 and August 2020, there were 113 patients referred to the specialized outpatient clinic, 88 of whom met the inclusion criteria and chose to participate after providing informed consent. Ten participants withdrew consent prior to completing the six-month treatment program, and eight declined to participate in the 12-month follow-up (online Supplementary Table S1). Nine patients (10.2%) completely discontinued antipsychotics and remained stable at the final follow-up, while an additional 24 (27.2%) reduced their antipsychotic dose by ⩾50%. Relapse was confirmed prior to drop-out in eight of the 18 dropouts, while 70 patients completed the six-month treatment and participated in the 12-month follow-up, yielding a total of 78 patients with known outcomes (Fig. 1).

**Figure 2.**
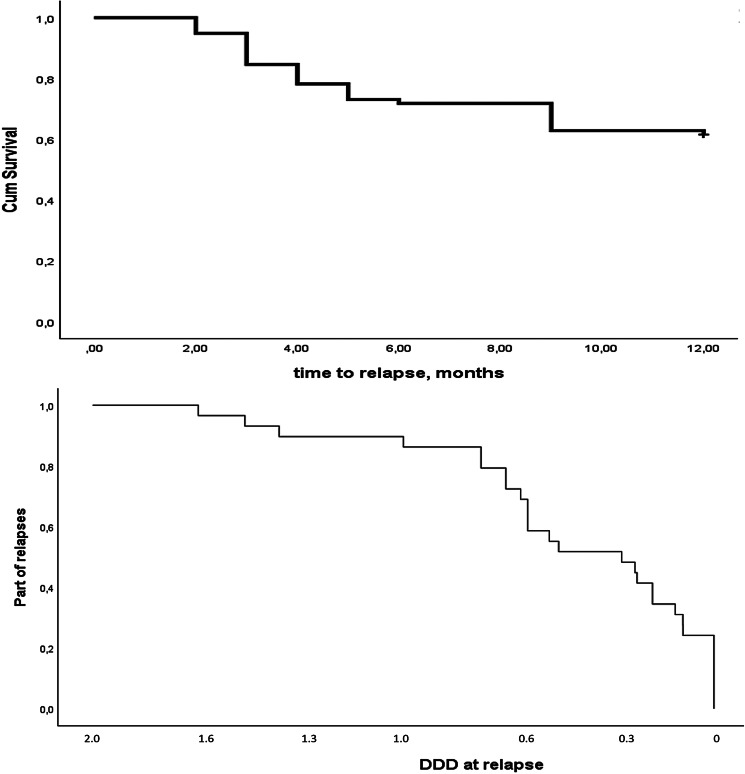
Survival curves. Upper curve shows time to relapse. Since the exact time for relapse is unknown at 6–12 months, it is arbitrarily defined as nine months. Lower curve shows relapse in relation to defined daily dose (DDD) at the time of relapse. The curve shows only few relapses at doses higher than 1 DDD, whereas half of the relapses are observed when doses are below 0.3 DDD.

By the end of the six-month period, 22 patients (27%) had experienced relapse, whereas this was the case for 29 (37%) patients after 12 months. Relapses were not restricted to a specific time period but occurred throughout the 12-month. Most relapses occurred at lower doses (Fig. 2a, b). Using Taipale et al.'s (2022) cut-offs, 23 of the 29 (86%) relapses coincided with an antipsychotic dose of < 0.9 DDD, while 17 of the 29 (59%) relapses occurred when the antipsychotic dose was < 0.6 DDD. Relapse was due to symptom worsening in 18 patients, hospitalization in 10, and functional decline in one. There were no suicidal or homicidal events during the 12-month observation period.

### Clinical measures

**Figure 3.**
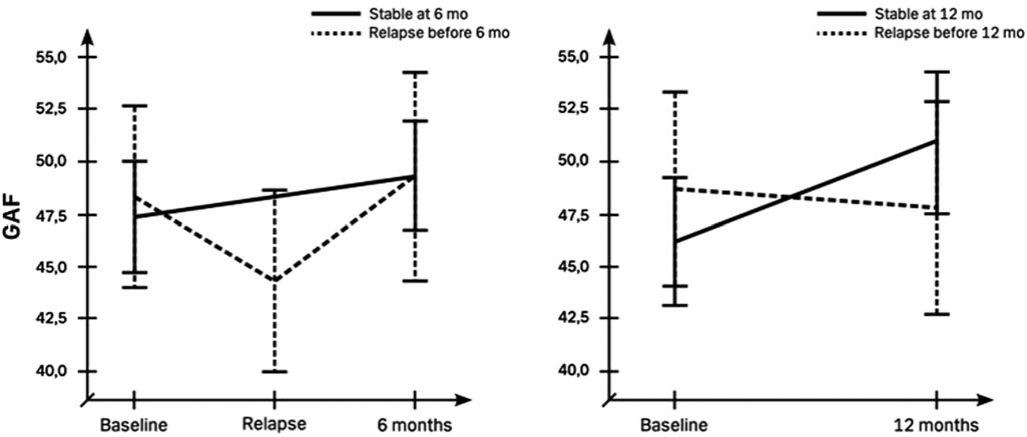
Level of function in terms of Global Assessment of Functioning (GAF) score divided based on stable patients and patients who relapsed after 6 and 12 months. Left graph illustrates mean GAF at baseline, lowest GAF in relation to relapse and GAF at six months. Right graph illustrates GAF at baseline and at 12 months follow up. GAF scores are missing for a few patients at follow-up.

Level of function improved in the whole group with 2.3 [Confidence interval 0.7–4.0] points on the GAF scale after six (*t*_70_ = 2.8, *p* = 0.006) and 3.3 [1.2–5.4] points after 12 months (*t*_62_ = 3.2, *p* = 0.002). Our analysis based on outcome showed that this was due to an improvement in stable patients of 2.4[0.6–4.3] points after 6 months (*t*_53_ = 2.7, *p* = 0.010) and 4.7[ 2.4–7.1] points after 12 months (*t*_43_ = 4.1, *p* < 0.001), whereas no significant change was observed in patients with relapse at 6 and 12 months (all *p* values > 0.3). Repeated measure ANOVA showed a trend effect of time (*F*_69,1_ = 3.4, *p* = 0.068) but no effect of group and no interaction after six months. Performing the same analysis with the lowest GAF score after relapse in patients who had experienced a relapse during the first six months, a group × time interaction was found (*F*_79,1_ = 11.5, *p* = 0.001), with a decrease in GAF score of 4 [1.3–6.8] points after registered relapse (*t*_21_ = 3.1, *p* = 0.005). Also, after 12 months a group × time interaction was observed (*F*_61,1_ = 6.6, *p* = 0.012) that still showed an increase in GAF score in patients who remained stable (*t*_43_ = 4.2, *p* < 0.001), but no change in patients experiencing relapse (Fig. 3).

PANSS total and all PANSS subscores were slightly lower in the whole group after six months (all *p* values < 0.04). An analysis divided up based on groups showed that this decrease was only significant for positive, general, and total scores in patients who remained stable (all *p* values < 0.003), whereas there were no changes between baseline and six months in patients experiencing a relapse during the period (all *p* values > 0.12). Repeated measure ANOVA showed no interaction, but an effect of time on total (*F*_71,1_ = 11.2, *p* = 0.001), positive (*F*_71,1_ = 8.0, *p* = 0.006) and general scores (*F*_71,1_ = 14, *p* < 0.001) and an effect of group on PANSS positive and general scores (*F*_71,1_ = 4 & 6.5–10, *p* = 0.049 & 0.01), which were higher at baseline and after six months in patients experiencing a relapse during the period (Table 1).

**Table 1.** Demographics at inclusion in study for all included patients and patients with known outcome status at 6 and 12 months

		All patients (*n* = 88)	Stable at 6 months (*n* = 59)		Relapse by 6 months (*n* = 22)	
		Baseline Mean (s.d.)	Baseline Mean (s.d.)	6 months Mean (s.d.)	Baseline Mean (s.d.)	6 months Mean (s.d.)
Age (years)		39.2 (11.2)	39.0 (12.0)		37.9 (11.2)	
Sex, female (%)		49 (56%)	35 (59%)		9 (41%)	
Age at diagnosis (years)^a^		25.5 (7.7)	24.1 (6.6)		28.4 (9.8)	
Duration of treatment (years)^b^		13.8 (9.6)	14.3 (9.8)		12.5 (9.2)	
CPZeq		404 (320)	429 (308)	219 (207)^1^	312 (293)	345 (263)
DDD		1.2 (0.7)	1.3 (0.69)	0.64 (0.50)^1^	0.98 (0.58)	0.99 (0.54)
GAF		46.7 (10.2)	47.1 (10.1)	48.5 (9.8)	48.3 (10.9)	50.9 (13.2)
UKU		17.9 (8.7)	17.2 (8.2)	10.5 (7.8)	20.7 (10.7)	13 (5.7)
DAI		0.1 (3.8)	0.27 (4.1)	0.45 (3.7)	−0.14 (3.4)	3.6 (3.1)^1^
PANSS	Positive	15.2 (5.5)	14.3 (5.0)	12.7 (4.7)^1^	16.8 (6.2)	16.2 (6.9)
	Negative	17.4 (6.1)	17.3 (5.8)	15.8 (5.3)	16.1 (6.0)	14.2 (5.9)
	General	31.8 (8.1)	30.1 (6.4)	25.8 (6.1)^1^	33.9 (9.0)	30.0 (8.9)
	Total score	64.4 (16.1)	61.7 (13.7)	54.3 (12.0)^1^	66.7 (18.2)	60.4 (17.0)
			Stable at 12 months (*n* = 49)		Relapse by 12 months (*n* = 29)	
			Baseline Mean (s.d.)	12 months Mean (s.d.)	Baseline Mean (s.d.)	12 months Mean (s.d.)
Age (years)			38.5 (12.4)		38.9 (10.7)	
Sex, female (%)			30 (61%)		13 (44.8%)	
Age at diagnosis (years)^c^			24.1 (6.6)		27.7 (9.7)	
Duration of treatment (years)^d^			14.2 (10.1)		12.5 (8.4)	
CPZeq			466 (323)	220 (201) ^1^	296 (259)	341 (294)
DDD			1.3 (0.72)	0.60 (0.50) ^1^	1.0 (0.56)	1.1 (0.53)
GAF			46.6 (10.5)	50.9 (11.6)^1^	48.6 (10.1)	47.7 (10.2)
PANSS	Positive		14.3 (4.9)		15.7 (6.0)	
	Negative		16.7 (6.0)		16.9 (5.5)	
	General		29.5 (6.0)		32.9 (8.6)	
	Total score		60.6 (13.3)		65.5 (16.8)	

SD, Standard Deviation; CPZeq, Clozapine equivalent; DDD, Defined Daily Doses; GAF, Global Assessment of Function; DAI, Drug Attitude Inventory; PANSS, Positive And Negative Symptom Scale.

a Information only available for 40 & 16 patients

b Information only available for 45 & 17 patients.

c Information only available for 35 & 19 patients.

d information only available for 36 & 23 patients.

1 significant change compared to baseline, *p* < 0.05.

### Antipsychotic medication

When entering the study, the mean antipsychotic dose was 409 mg (±306) CPZeq. Second generation antipsychotics was tapered for 60 patients (68%), 11 patients (13%) tapered first generation antipsychotics and 17 patients (19%) tapered clozapine. Long acting injectables was received by 18 patients (20%), and antipsychotic polypharmacy was received by 14 patients (16%). After six months, there was a decrease in antipsychotic dose of 159 [122–196] mg CPZeq to 250 mg (±227) (*Z*_78_ = 6.4, *p* < 0.001). This mean dose remained stable by 12 months 255 mg (±236). The analysis divided up based on group showed that the decrease was only significant in patients who remained stable (*Z*_61&49_ = 6.7 & 6.1, *p* < 0.001), whereas no significant change in antipsychotic dose was observed in patients experiencing a relapse after six or 12 months.

Among patients in the relapse group, six patients received an increased dose after six and 12 months, whereas the rest either remained on medication equivalent to the dose at inclusion (*n*_6_ = 9 and *n*_12_ = 8) or became stabilized on a decreased dose compared to the baseline dose (*n*_6_ = 4 and *n*_12_ = 6). It must be noted, however, that data are missing on medication dose for three patients with known relapse after six months, and nine patients with known relapse after 12 months (Table 1).

For the whole study group, we saw an improvement of 7 [4.6–9.2] points in the rating of side effects after six months as measured with the total UKU score, the improvement was significant for each of the UKU subscales (*Z*_78,2_ = 3.4–6.2, all *p* < 0.001). Analysis divided up based on groups showed that patients who tolerated the reduced dose without clinical worsening improved in total score and on all subscales (*Z*_59,2_ = 3.0–5.2, all *p* < 0.003), whereas patients with relapse improved in total score and only in the psychiatric, autonomic, and other symptoms subscales (*Z*_19,2_ = 2.3–3.4 all *p* < 0.02), but not significantly on neurological symptoms and sexual complaints (*Z*_19,2_ = 1.4 & 1.5 *p* = 0.16 & 0.14)

The attitude toward antipsychotics as measured by DAI improved at trend level for the whole group after six months (*Z*_74_ = 1.88, *p* = 0.06). Analysis separated based on outcome showed that improvement was only observed in patients who experienced a relapse (*Z*_16_ = 3.1, *p* = 0.002) and not in stable patients (*p* = 0.65). After six months, patients with a relapse rated higher on DAI than stable patients (*Z*_46_ = 3.0, *p* = 0.002), whereas the DAI-score at baseline (*p* = 0.46) showed no differences.

### Predictive analyses

Relapse during the first six months was associated with higher baseline PANSS general score, lower PANSS negative scores and lower baseline medication dose (CPZeq) at baseline, although not contributing significantly. Variables removed were GAF, PANSS positive, and age (Table 2). Running the analysis again with years since first antipsychotic treatment (*N* = 61) did not change the significantly contributing variables. Including age at diagnoses changed the final result to a model also including age and age at diagnosis. Higher age at first schizophrenia diagnosis and lower age at time of study participation were associated with increased risk of relapse.

**Table 2.** The contribution of the independent variables in each of the final backward logistic regression models predicting relapse status after 6 or 12 months

Model	Predictors	*β*	s.e.	*Z* Wald	*p* > |z|
**1**	**Relapse 6 month, model explained 22% of the variance (Nagelkerke R2, *N* = 80, *p* = 0.005)**				
	**PANSS General**	0.14	0.05	8.3	0.004
	**PANSS Negative**	−0.15	0.07	5.1	0.025
	**CPZ-eq baseline**	−0.002	0.001	2.7	0.106
	**GAF, age, PANSS pos**	removed			
**2**	**Relapse 6 month, model explained 43% of the variance (Nagelkerke R2, *N* = 55, *p* = 0.001)**				
	**PANSS General**	0.20	0.08	6.9	0.018
	**PANSS Negative**	−0.27	0.10	7.0	0.019
	**Age**	−0.08	0.04	3.0	0.086
	**Age at diagnoses**	0.13	0.07	4.2	0.039
	**CPZeq**	−0.003	0.001	3.0	0.086
	**GAF, PANSS pos**	removed			
**3**	**No significance of adding years since first antipsychotic treatment**				
**4**	**Relapse 12 month, model explained 27% of the variance (Nagelkerke R2, *N* = 72, *p* = 0.001)**				
	**PANSS General**	0.14	0.05	8.5	0.004
	**GAF**	0.08	0.03	5.6	0.018
	**CPZ-eq baseline**	−0.003	0.001	6.2	0.013
	**PANSS neg, PANSS pos, age**	removed			
**5-6**	**No effect of adding age at diagnoses or years since first antipsychotic treatment**				

Relapse at 12 months was associated with lower baseline CPZeq, higher PANSS general, and higher GAF score at baseline. Running the analysis again with age at diagnosis or years since first treatment did not improve the final model.

## Discussion

In this cohort study, we show that 63% of the patients with schizophrenia were able to reduce antipsychotic dose over a six-month period and remain stable at follow-up after 12 months. For these patients, mean CPZeq was reduced from 466 to 220 mg. They also experienced a small improvement in level of functioning. While this observation is in line with the results other studies reported (Harrow et al., 2017; Wunderink et al., 2013), a recent randomized trial did not find an improvement in level of function in patients randomized to dose reduction (Moncrieff et al., 2023). That trial did however not report on stable patients *v.* patients who relapsed. Together, these data suggest that level of function mainly improves in patients who remain stable on lower doses. Although the average improvement of 4 points on the GAF scale is relatively small and may perhaps seem insignificant to an observer, it may still be important for the patients. Being less socially isolated may for some patients lead to further improvement over time. In addition, these patients experienced a reduction in adverse effects, which for most patients were the main motivational factor for seeking out the tapering clinic (Nøstdal, Hilker, Nielsen, & Nielsen, 2024).

A relative worsening of existing, or re-emergence of previous, psychotic symptoms was observed in 37% of patients. While this unfortunately resulted in admittance to a psychiatric ward for 10 cases (13%), we also observed that several patients who experienced relapse were able to restabilize, with symptom severity and level of functioning returning to baseline values as measured by PANSS and GAF, respectively. This is in line with the observations that most patients respond to reintroduction of antipsychotic medication after a relapse (Emsley, Chiliza, Asmal, & Harvey, 2013b). Importantly, the majority experienced stabilization of symptoms at a lower dose of antipsychotics compared to what they received at baseline, achieved either through reduced dose or switching to an alternative drug, resulting in a minor yet significant reduction in experienced side effects. Also, there were no incidents of suicidality or acts of violence, and none of the patients developed treatment resistance to antipsychotic medication following relapse. However, for a few individuals who experienced a relapse, follow-up data are missing, which is why we do not know if these patients belong to the well-documented subgroup with more severe relapses who may develop treatment failure (Emsley, Chiliza, & Asmal, 2013a; Emsley, Nuamah, Hough, & Gopal, 2012). While the sample size is relatively small and the observation period relatively short, we postulate that the gradual reduction of antipsychotics in a safe and supportive environment can mitigate the risk of the severe adverse events normally associated with discontinuation of antipsychotics when done abruptly and/or independently. Further studies are warranted to test this hypothesis, preferably with larger sample size and with longer follow-up periods. Also, the possible effect of increased psychological and social support during the tapering period needs to be explored.

Surprisingly, mean PANSS score decreased after six months in both stable and relapsed patients. We have speculated whether the close contact and frequent PANSS interviews with the same staff may have affected reporting and registration of symptoms, despite our effort to avoid this by regular supervised ratings in groups. Other explanations may be that six months PANSS data are missing for a few relapsed patients, and these may be hospitalized patients who have not yet stabilized after their relapse. In the stable patients, it was reported that the tapering process gave them a feeling of increased empowerment which made them feel less behavioral affected by their symptoms (Mølgaard, Nielsen, Roed, & Nielsen, 2024).

Interestingly, we observed a significant improvement in the attitude toward antipsychotics of patients who relapsed following stabilization after relapse. We interpret this change as a valuable insight for the individual patient, demonstrating for them a positive effect of their current treatment, which could aid future decision making for continuing treatment. We have previously reported uncertainty in the necessity of antipsychotic treatment as a major motivational factor for seeking antipsychotic tapering or discontinuation (Nøstdal et al., 2024). The observed change in attitude toward antipsychotics might reflect clarification of the question: ‘Do I still need antipsychotic treatment?’ or, in the case of patients experiencing adverse effects from the ongoing treatment: ‘Does the beneficial effect of treatment outweigh the cost of adverse effects?’

Our tapering protocol was rather quick compared to Liu et al.'s (2023), and our relapse rate was also higher. Relapses were primarily observed when lower doses of antipsychotic medication were given, i.e. under 0.9 DDD. This might indicate that physicians should apply extra care when guiding their patients through the last part of the dose reduction and the observation supports the hyperbolic approach that Horowitz, Murray, and Taylor (2021) suggested. Future tapering protocols may benefit from a more rapid initial dose reduction, followed by gradually slowing down frequency and amount reduced per interval as the antipsychotic dose approaches 0.9 DDD, and possibly slowing down even further by 0.6 DDD. Especially for patients who received higher doses or had longer treatment duration it has been recommended to extend the tapering period over several years to allow the brain to re-adapt incrementally to lower levels of antagonism (Horowitz et al., 2022). Another challenge we faced during the final part of dose reduction was the limited availability of low dose tablets. This problem could have been solved by introducing the use of liquid formulations when possible.

Compared to the RADAR trial also on non-remitted patients, our cohort had more symptoms and received a higher baseline CPZeq dose which was tapered more quickly. All three factors may have contributed to the higher one-year relapse rate in our study. In our attempt to identify predictive factors, we included clinical measures previously shown to be associated with successful discontinuation from antipsychotic treatment (Tani et al., 2018). In line with previous findings, we found that higher level of PANSS general symptoms at baseline was associated with increased risk of relapse. This may suggest that special attention should be given to the sickest patients during tapering, as there may be some beneficial effect of the medication even though patients are still symptomatic and may doubt this themselves. In contrast to some previous findings (Tani et al., 2018) we found that older age at onset of illness and lower antipsychotic dose at baseline predicted higher risk of relapse in this cohort. However, higher age at first schizophrenia diagnosis and lower age at time of study were only associated with relapse at six months. As a result, we can speculate whether this indicates that short duration of illness/antipsychotic treatment is associated with a higher risk for a rapid destabilization. A previous review did not find this association; however, they focused on first-episode patients, while our cohort primarily consisted of patients with a much longer duration of illness (Emsley et al., 2013b).

A clear limitation arises due to the built-in timeframe in our study design, which has a relatively short intervention period (six months) and subsequent observation period (12-month follow-up). It is likely that some of the patients defined as stable are still in a ‘honeymoon phase’ at the final observation point and might suffer relapse in the following months, resulting in false positive cases, which would naturally affect the predictive models. The relatively short observation period was the result of a compromise between competing interests, where a longer observation period was pursued from a research standpoint, while the state grant requested a higher number of treated patients within the project's same overall timeframe. The experiences and data collected have, nevertheless, enabled the expansion of the tapering clinic to include future study protocols with longer treatment and observation timeframes.

Another limitation is that the very nature of the specialized outpatient clinic and referral process likely resulted in selection bias. Deprescribing is regularly done in Danish outpatient clinics (Stürup et al., 2022a), and the referred population might represent complex patients who the referring clinic did not feel comfortable assisting in deprescribing; or the population may comprise more resourceful patients capable of seeking alternative treatment options due to hesitancy from the regular clinic.

With the above limitations in mind, we still argue that our study offers important information by showing interesting trends toward improved level of functioning and reduced adverse effects from the medication among those whose antipsychotic dose was successfully reduced and a low risk of severe adverse events. Most patients experiencing a relapse had a relatively swift re-stabilization with the added benefit of an improved attitude toward antipsychotics following relapse, hypothetically improving the treatment alliance and future adherence. The hope is that future studies with a larger sample size and increased statistical power will reveal better predictive models.

## Supporting information

Nøstdal et al. supplementary material Nøstdal et al. supplementary material

## Supplementary material

The supplementary material for this article can be found at https://doi.org/10.1017/S0033291724001910

## References

[ref1] Association, A. P. (1987). Diagnostic and statistical manual of mental disorders: DSM-III R (4th ed.). Washington, DC: American Psychiatric Association, [1994] ©1994. Retrieved from https://search.library.wisc.edu/catalog/999733358502121

[ref2] Beasley, C. M., Sutton, V. K., Hamilton, S. H., Walker, D. J., Dossenbach, M., Taylor, C. C., … Tollefson, G. D. (2003). A double-blind, randomized, placebo-controlled trial of olanzapine in the prevention of psychotic relapse. Journal of Clinical Psychopharmacology, 23(6), 582–594. 10.1097/01.jcp.0000095348.32154.ec 14624189

[ref3] Bowtell, M., Ratheesh, A., McGorry, P., Killackey, E., & O'Donoghue, B. (2018). Clinical and demographic predictors of continuing remission or relapse following discontinuation of antipsychotic medication after a first episode of psychosis. A systematic review. Schizophrenia Research, 197, 9–18. 10.1016/j.schres.2017.11.010 29146020

[ref4] Chen, E. Y. H., Hui, C. L. M., Lam, M. M. L., Chiu, C. P. Y., Law, C. W., Chung, D. W. S., … Honer, W. G. (2010). Maintenance treatment with quetiapine versus discontinuation after one year of treatment in patients with remitted first episode psychosis: Randomised controlled trial. BMJ *(*Online*)*, 341(7770), 8–9. 10.1136/bmj.c4024 PMC2924475 20724402

[ref5] Correll, C. U., Rubio, J. M., & Kane, J. M. (2018). What is the risk-benefit ratio of long-term antipsychotic treatment in people with schizophrenia? World Psychiatry, 17(2), 149–160. 10.1002/wps.20516 29856543 PMC5980517

[ref7] Emsley, R., Chiliza, B., & Asmal, L. (2013a). The evidence for illness progression after relapse in schizophrenia. Schizophrenia Research, 148(1–3), 117–121. 10.1016/j.schres.2013.05.016 23756298

[ref8] Emsley, R., Chiliza, B., Asmal, L., & Harvey, B. H. (2013b). The nature of relapse in schizophrenia. BMC Psychiatry, 13, 1–8. 10.1186/1471-244X-13-50 23394123 PMC3599855

[ref6] Emsley, R., Nuamah, I., Hough, D., & Gopal, S. (2012). Treatment response after relapse in a placebo-controlled maintenance trial in schizophrenia. Schizophrenia Research, 138(1), 29–34. 10.1016/j.schres.2012.02.030 22446143

[ref9] Ernst, R., Lindström, E., Nielsen, J., & Levander, S. (2012). DAI-10 is as good as DAI-30 in schizophrenia. European Neuropsychopharmacology, 22(10), 747–750. 10.1016/j.euroneuro.2012.02.008 22440974

[ref10] Gupta, S., Cahill, J. D., & Miller, R. (2018). Deprescribing antipsychotics: A guide for clinicians. BJPsych Advances, 24(5), 295–302. 10.1192/bja.2018.2

[ref11] Harrow, M., Jobe, T. H., Faull, R. N., & Yang, J. (2017). A 20-year multi-followup longitudinal study assessing whether antipsychotic medications contribute to work functioning in schizophrenia. Psychiatry Research, 256, 267–274. 10.1016/j.psychres.2017.06.069 28651219 PMC5661946

[ref12] Hogan, T. P., Awad, A. G., & Eastwood, R. (1983). A self-report scale predictive of drug compliance in schizophrenics: Reliability and discriminative validity. Psychological Medicine, 13(1), 177–183. 10.1017/S0033291700050182 6133297

[ref13] Højlund, M., Kemp, A. F., Haddad, P. M., Neill, J. C., & Correll, C. U. (2021). Standard versus reduced dose of antipsychotics for relapse prevention in multi-episode schizophrenia: A systematic review and meta-analysis of randomised controlled trials. The Lancet Psychiatry, 8(6), 471–486. 10.1016/S2215-0366(21)00078-X 34023019

[ref15] Horowitz, M. A., Moncrieff, J., de Haan, L., Bogers, J. P. A. M., Gangadin, S. S., Kikkert, M., … Sommer, I. E. C. (2022). Tapering antipsychotic medication: Practical considerations. Psychological Medicine, 52(1), 32–35. 10.1017/S0033291721003299 34542027

[ref14] Horowitz, M. A., Murray, R. M., & Taylor, D. (2021). Tapering antipsychotic treatment. JAMA Psychiatry, 78(2), 125–126. 10.1001/jamapsychiatry.2020.21 32777027

[ref16] Kay, S. R., Fiszbein, A., & Opler, L. A. (1987). The positive and negative syndrome scale (PANSS) for schizophrenia. Schizophrenia Bulletin, 13(2), 261–276. 10.1093/schbul/13.2.261 3616518

[ref17] Leucht, S., Samara, M., Heres, S., Patel, M. X., Woods, S. W., & Davis, J. M. (2014). Dose equivalents for second-generation antipsychotics: The minimum effective dose method. Schizophrenia Bulletin, 40(2), 314–326. 10.1093/schbul/sbu001 24493852 PMC3932104

[ref18] Lingjærde, O., Ahlfors, U. G., Bech, P., Dencker, S., & Elgen, K. (1987). The UKU side effect rating scale: A new comprehensive rating scale for psychotropic drugs and a cross-sectional study of side effects in neuroleptic-treated patients. Acta Psychiatrica Scandinavica, 76(s334), 100. 10.1111/j.1600-0447.1987.tb10566.x 2887090

[ref19] Liu, C. C., Hsieh, M. H., Chien, Y. L., Liu, C. M., Lin, Y. T., Hwang, T. J., … Hwu, H. G. (2023). Guided antipsychotic reduction to reach minimum effective dose (GARMED) in patients with remitted psychosis: A 2-year randomized controlled trial with a naturalistic cohort. Psychological Medicine, 53(15), 7078–7086. 10.1017/S0033291723000429 36896797 PMC10719630

[ref20] Mccormack, J., & Elwyn, G. (2018). Shared decision is the only outcome that matters when it comes to evaluating evidence-based practice. BMJ Evidence-Based Medicine, 23(4), 137–139. 10.1136/bmjebm-2018-110922 30002077

[ref21] Moilanen, J., Haapea, M., Miettunen, J., Jääskeläinen, E., Veijola, J., Isohanni, M., & Koponen, H. (2013). Characteristics of subjects with schizophrenia spectrum disorder with and without antipsychotic medication - A 10-year follow-up of the Northern Finland 1966 Birth Cohort study. European Psychiatry, 28(1), 53–58. 10.1016/j.eurpsy.2011.06.009 21920710

[ref22] Mølgaard, S. N., Nielsen, M. Ø., Roed, K., & Nielsen, J. (2024). Clinical experiences of guided tapering of antipsychotics for patients with schizophrenia– a case series. BMC Psychiatry, 24(1), 0–8. 10.1186/s12888-024-05699-y PMC10981298 38553687

[ref23] Moncrieff, J., Crellin, N., Stansfeld, J., Cooper, R., Marston, L., Freemantle, N., … Priebe, S. (2023). Antipsychotic dose reduction and discontinuation versus maintenance treatment in people with schizophrenia and other recurrent psychotic disorders in England (the RADAR trial): An open, parallel-group, randomised controlled trial. The Lancet Psychiatry, 10(11), 848–859. 10.1016/S2215-0366(23)00258-4 37778356

[ref24] Munkholm, K., Horowitz, M. A., & Moncrieff, J. (2022). Maintenance antipsychotic trials and the effect of withdrawal. The Lancet, 400(10357), 995. 10.1016/S0140-6736(22)01467-2 36154688

[ref25] Nøstdal, A., Hilker, R., Nielsen, J., & Nielsen, M. Ø. (2024). Motivations for and experiences with antipsychotic tapering among patients with schizophrenia seeking guided dose reduction. Psychiatric Services, in press. 10.1176/appi.ps.20230641 39482959

[ref26] Potla, S., Al Qabandi, Y., Nandula, S. A., Boddepalli, C. S., Gutlapalli, S. D., Lavu, V. K., … Hamid, P. (2023). A systematic review of the need for guideline recommendations; slow tapering vs. maintenance dose in long-term antipsychotic treatment: 2022. Cureus, 15(2), e34746. 10.7759/cureus.34746 36777974 PMC9904861

[ref27] Ramspek, C. L., Steyerberg, E. W., Riley, R. D., Rosendaal, F. R., Dekkers, O. M., Dekker, F. W., & van Diepen, M. (2021). Prediction or causality? A scoping review of their conflation within current observational research. European Journal of Epidemiology, 36(9), 889–898. 10.1007/s10654-021-00794-w 34392488 PMC8502741

[ref28] Rey, M. J., Schulz, P., Costa, C., Dick, P., & Tissot, R. (1989). Guidelines for the dosage of neuroleptics. I: Chlorpromazine equivalents of orally administered neuroleptics. International Clinical Psychopharmacology, 4(2), 95–104. 2568378 10.1097/00004850-198904000-00001

[ref29] Scott, I. A., Hilmer, S. N., Reeve, E., Potter, K., Le Couteur, D., Rigby, D., … Martin, J. H. (2015). Reducing inappropriate polypharmacy: The process of deprescribing. JAMA Internal Medicine, 175(5), 827–834. 10.1001/jamainternmed.2015.0324 25798731

[ref30] Statistics Denmark. (2018). Population at the first day of the quarter by age, sex, region and time (2018Q1). Retrieved from https://www.statbank.dk/INDAMP01

[ref31] Stürup, A. E., Hjorthøj, C., Albert, N., Dolmer, S., Birk, M., Ebdrup, B. H., … Nordentoft, M. (2022a). Tapered discontinuation vs. maintenance therapy of antipsychotic medication in patients with first-episode schizophrenia: Obstacles, findings, and lessons learned in the terminated randomized clinical trial TAILOR. Frontiers in Psychiatry, 13, 910703. 10.3389/fpsyt.2022.910703 35935409 PMC9355082

[ref32] Stürup, A. E., Nordentoft, M., Jimenez-Solem, E., Osler, M., Davy, J. W., Christensen, T. N., … Hjorthøj, C. (2022b). Discontinuation of antipsychotics in individuals with first-episode schizophrenia and its association to functional outcomes, hospitalization and death: A register-based nationwide follow-up study. Psychological Medicine, 53(11), 5033–5041. 10.1017/s0033291722002021 35818718

[ref33] Taipale, H., Tanskanen, A., Luykx, J. J., Solmi, M., Leucht, S., Correll, C. U., & Tiihonen, J. (2022). Optimal doses of specific antipsychotics for relapse prevention in a nationwide cohort of patients with schizophrenia. Schizophrenia Bulletin, 48(4), 774–784. 10.1093/schbul/sbac039 35524479 PMC9212108

[ref34] Tani, H., Suzuki, T., Wolfgang Fleischhacker, W., Tomita, M., Mimura, M., & Uchida, H. (2018). Clinical characteristics of patients with schizophrenia who successfully discontinued antipsychotics: A literature review. Journal of Clinical Psychopharmacology, 38(6), 582–589. 10.1097/JCP.0000000000000959 30300291

[ref35] Taylor, D., & Horowitz, M. A. (2020). Antipsychotics and mortality – more clarity needed. Psychological Medicine 50(16), 2814–2815. 10.1017/S0033291720004535 33261685

[ref36] Tiihonen, J., Tanskanen, A., & Taipale, H. (2018). 20-year nationwide follow-up study on discontinuation of antipsychotic treatment in first-episode schizophrenia. American Journal of Psychiatry, 175(8), 765–773. 10.1176/appi.ajp.2018.17091001 29621900

[ref37] Tiihonen, J., Taipale, H., & Correll, C. U. (2020). Commentary on Robert Whitaker's viewpoint. Psychological Medicine, 50(16), 2653–2654. 10.1017/S0033291720003591 33100236

[ref38] Velligan, D. I., Sajatovic, M., Hatch, A., Kramata, P., & Docherty, J. P. (2017). Why do psychiatric patients stop antipsychotic medication? A systematic review of reasons for nonadherence to medication in patients with serious mental illness. Patient Preference and Adherence, 11, 449–468. 10.2147/PPA.S124658 28424542 PMC5344423

[ref39] Whitaker, R. (2020). Viewpoint: Do antipsychotics protect against early death? A critical view. Psychological Medicine, 50(16), 2643–2652. 10.1017/S003329172000358X 33050955

[ref40] WHO. (2021). ATC/DDD N05A Antipsychotics. Retrieved December 11, 2022, from 2021-12-14 website https://www.whocc.no/atc_ddd_index/?code=N05A&showdescription=no

[ref41] Wils, R. S., Gotfredsen, D. R., Hjorthøj, C., Austin, S. F., Albert, N., Secher, R. G., … Nordentoft, M. (2017). Antipsychotic medication and remission of psychotic symptoms 10 years after a first-episode psychosis. Schizophrenia Research, 182(2017), 42–48. 10.1016/j.schres.2016.10.030 28277310

[ref42] Wunderink, L., Nieboer, R. M., Wiersma, D., Sytema, S., & Nienhuis, F. J. (2013). Recovery in remitted first-episode psychosis at 7 years of follow-up of an early dose reduction/discontinuation or maintenance treatment strategy long-term follow-up of a 2-year randomized clinical trial. JAMA Psychiatry, 70(9), 913–920. 10.1001/jamapsychiatry.2013.19 23824214

[ref43] Yoshida, K., & Takeuchi, H. (2021). Dose-dependent effects of antipsychotics on efficacy and adverse effects in schizophrenia. Behavioural Brain Research, 402(October 2020), 113098. 10.1016/j.bbr.2020.113098 33417992

