# Supplementary material for: Deprescribing antipsychotics in patients with schizophrenia: findings from a specialized clinic
Source: Psychol Med. 2024 Oct 15;54(13):3697–706. doi: 10.1017/S0033291724001910 (PMC11536106; doi:10.1017/S0033291724001910)

**Supplement material**

**Supplementary table 1. Demographics at inclusion in study, by drop out-status at 12 months**

|  | | **Missing (n = 10)** |
| --- | --- | --- |
|  | | **Mean (SD)** |
| Age (years) | | 43.7 (9.7) |
| Sex, female (%) | | 6 (60%) |
| Age at diagnosis | | 26.5 (5.2) |
| Duration of treatment | | 15.8 (12.4) |
| CPZeq | | 411 (420) |
| DDD | | 1.3 (0.97) |
| PANSS | Positive | 18.3 (6.3) |
|  | Negative | 22.9 (6.1) |
|  | General | 40.2 (10.7) |
|  | Total score | 81.4 (18.4) |
| GAF | | 38.2 (4.4) |
| DAI | | -2.33 (2.69) |

**Supplementary table 2. Examples of tapering regimens, * marks relapse at that point**

| **Compound** | **Dose at baseline** | **1st month** | **2nd month** | **3rd month** | **4th month** | **5th month** | **6th month** |
| --- | --- | --- | --- | --- | --- | --- | --- |
| **Aripiprazole** | 15 | 10 | 10 | 10 | 5 | 5 | 0 |
| **Aripiprazole** | 5 | 2.5 | 2.5 | 2.5 | 0 | 0 | 0* |
| **Aripiprazole LAI** | 400 | 360 | 320 | 280 | 240 | 200 | 180 |
| **Olanzapine** | 20 | 17.5 | 15 | 12.5 | 12.5 | 12.5 | 10 |
| **Olanzapine** | 10 | 7.5 | 5 | 5 | 2.5 | 2.5. | 0 |
| **Risperidone** | 2 | 1.5 | 1 | 0.5 | 0.5 | 0 | 0 |
| **Paliperidone LAI** | 75 | 50 | 50 | 50 | 25 | 25 | 25* |
| **Lurasidone** | 111 | 92,5 | 92,5 | 74 | 55,5 | 37 | 37 |
| **Quetiapine** | 600 | 550 | 500 | 450 | 400 | 350 | 300 |
| **Clozapine** | 400 | 362,.5 | 325 | 287,5 | 250 | 215,5 | 175 |
| **Perphenazine** | 6 | 4 | 4 | 2 | 2 | 2 | 0 |
| **Zuclopenthixol** | 8 | 6 | 6 | 4 | 4 | 2 | 2 |
| **Zuclopenthixol LAI** | 100/14d | 90/14d | 80/14d | 70/14d | 70/14d | 60/14d | 50/14d |

**Supplement figures showing Global Assessment of Functioning score (GAF) over time**

**Figure 1. GAF for 17 of the 22 patients who had been through a relapse at 6 moths follow up**.

Here individual trajectories are illustrated for GAF at baseline, lowest GAF at/ after relapse and at six months follow up.

**
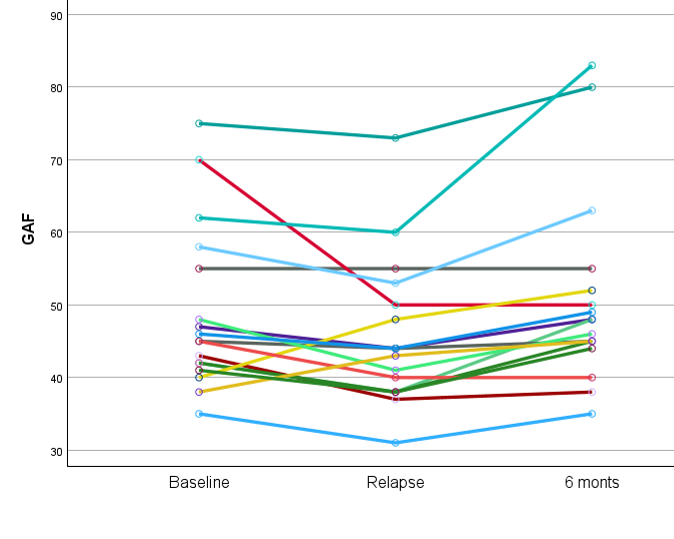
**

**Figure 2 GAF for 53 of the 59 patients who were stable at 6 moths follow up.**

**
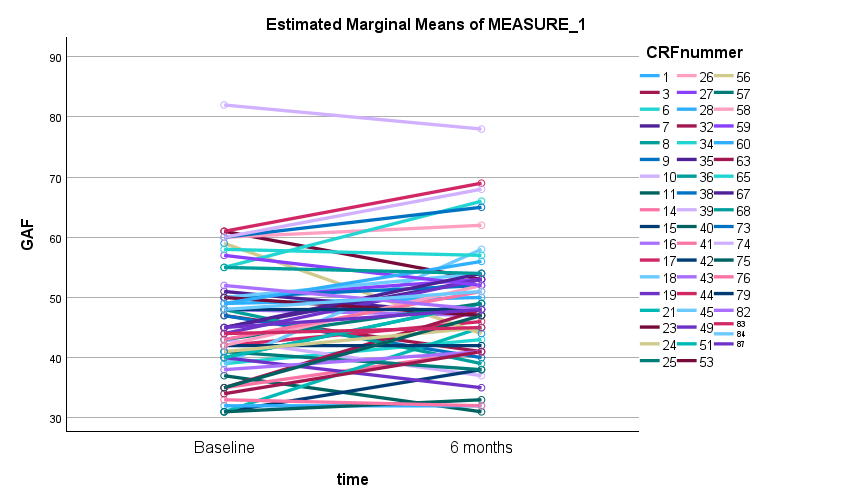
**

**Figure 3 GAF for 18 of the 29 patients who had experienced relapse before 12 moths follow up.**


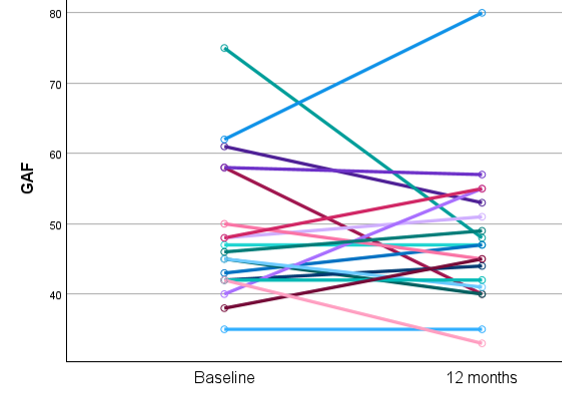


**Figure 4 GAF for 43 of the 49 patients who were stable at 12 moths follow up.**


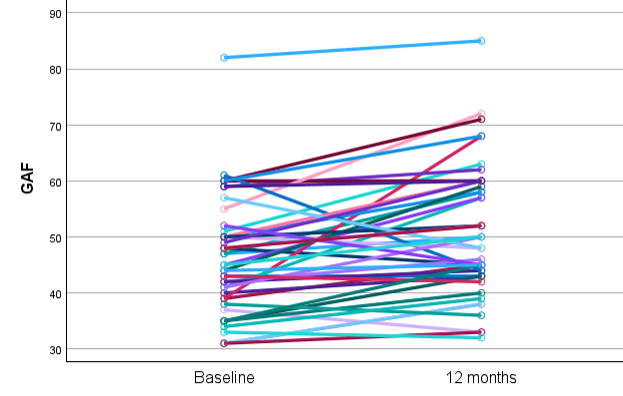

Supplement: Nøstdal et al. supplementary material [file S0033291724001910sup001.docx]
